# Exploring the factors affecting violence among Iranian male adolescents

**DOI:** 10.1186/s12889-022-14710-8

**Published:** 2022-11-30

**Authors:** Tahereh Solimannejad, Marziyeh Ebrahimi, Mohamad Solimannejad

**Affiliations:** 1 Alborz University of Medical Sciences, Karaj, Iran; 2 Shahid Beheshti University, Tehran, Iran

**Keywords:** Adolescence, Violence, Educational environment, Family, Peers, Social environment

## Abstract

Increasing violence behaviors among high school adolescents affects different dimensions of the efficiency of educational institutions. The present qualitative research was conducted in 2021 to explain violence behaviors among male adolescents in Karaj, Iran. Twenty senior high school students were selected using purposive sampling with maximum variation. Individual semi-structured interviews conducted based on the participants’ experiences were analyzed in MAXQDA 2020. According to the model proposed by Strauss and Corbin, causal conditions comprised “economic challenges facing the family”, “academic apathy”, “seeking pleasure”, “self-other differentiation” and “family disorganization”, contextual conditions included “school inefficiency”, “environmental conditions” and “cultural diversity” and confounding conditions consisted of “peer pressure” and “puberty”. The strategies of adolescents with violent behaviors such as ignoring school rules and bullying can cause their humiliation, loss of ideal opportunities in life, punishment and rejection. “Violence as the collapse of individual and socio-familial capital” was labeled the core category of the present study. According to the present findings, violence in adolescents is affected by a multilevel mechanism based on social relationships. As a major obstacle to educational objectives, the violence emerging at school as a reliable and safe environment can spread to other social domains in the long run.

## Problem statement

As a major social harm among adolescents, violence is prevalent among school students [1]. High-risk and violent behaviors have been reported in 19.7% of male and 19.6% of female Iranian students [2]. The UNICEF (2019) found almost 32% of children to suffer violence at least once a month in most schools across the world [3]. The WHO defined violence in children and adolescents as any actions or failure to act that damage their health, well-being, psychophysical integrity, freedom and right to achieve full development. Violence exerts serious and lifelong effects on psychophysical health and social functioning. In 2020, the WHO reported 200,000 murders worldwide among the youth and adolescents aged 10–29 years [4]. Violence is an action that is deliberately taken by any individuals to harm another person at any place and time [5]. Violence behaviors in school is related to the interpersonal type and refers to any kinds of voluntary misconduct such as mental and sexual harassment, physical harm, threatening or abuse. Effective variables in the complex phenomenon of school violence include impulsivity, empathy, attitudes toward violence, external and internal behaviors, self-efficacy, anxiety, depression, drug abuse and parenting styles [6–8]. Thus, the presence of one or some of these factors can pave the way for violent behavior among the adolescents.

Safety and peace at schools are essential for learning and gaining positive experiences in the developmental process of children and adolescents [9]. According to the social disorganization theory, physical and social environments influence selective behaviors. Criminal behaviors are more likely to occur in individuals facing crimes or violence. Media play key roles in encouraging individuals to commit crimes through institutionalizing cultural and behavioral norms [10]. Communication skills were found to relate to human communication factors, training programs, familial factors, school factors and violence [11]. A qualitative study extracted road accidents, falls, quarrels, sexual violence, robbery and vandalism as common factors among Kenyan adolescents. Individual factors such as gender, poverty, drug use, parental behaviors and school dropout were also found to be correlated with certain factors at broader levels such as insecure neighborhood and livelihood sources at risk [12].

Despite the positive and constructive functions of educational entities, schools constitute environments for committing violent behaviors, which are mainly institutionalized in individuals through social learning. According to the social learning theory, individuals tend to engage in delinquent behaviors in the face of attitudes that justify and encourage violation of the law [13] and individuals learn violent behaviors within their living sociocultural context [14]. The results of the study by Brazil-Murray (2018) have indicated that being connected with each other through social networks can lead to the feelings of connectedness and relatedness, something that is the code by social learning theory [15].

School violence lowers self-esteem, self-efficacy and academic performance in students [16] and spoils their talent and poses psychophysical and social health hazards to communities. According to social control theory, individuals follow norms and rules, as community-related control prevents crime [17]. Hamama and Ronnen-Shenhaw [18] found higher frequencies of violence in divorced parents, young people and boys compared to in girls. Social support is an environmental and self-control resource that reduces violent behaviors.

According to Vazina et al. [19], domestic violence, inappropriate socialization, low levels of parental understanding, poor parental supervision, family tensions and conflicts, wicked and deviant peers and high-risk behaviors significantly affect violence in adolescence and early adulthood.

In regards to the focus of the study, most of the studies on violence behaviors are related to violence against women and less has been done to explore violence among Iranian adolescents while the available statistics show that at least 20% of Iranian students experience some type of violence in school [2].

As an immigrant-friendly city in Iran with a business atmosphere, Karaj demographically comprises different subcultures and ethnic groups of Iranians and non-Iranians. This cultural and demographic heterogeneity can cause social conflicts.

Qualitative research helps identify the causes and foundations of violence behaviors in male students. Given violent behaviors among students as a growing issue in schools, determining these causes can assist in finding effective context in violence.

## Categories of violent behaviors

Violent behaviors are categorized as physical, e.g. pushing, fisting, stabbing, psychological/emotional, e.g., verbal insults, threats, harsh looks, relational/social (rumor) and cyber (sending texts or photos) in type. As the most common and objective type of bullying, physical violence includes behaviors such as hitting and pushing. Psychological/emotional violence involves insults and threats that aim at the accusation, humiliation or intimidation of the victim. Rumors are spread in relational/social violence to socially eliminate the victim. This type of violence is relatively rare and subtly used to damage the social relationships of the victim with their classmates. As a recently-identified type of violence, cyber-violence or cyberbullying aims at threatening the victims by spreading rumors or sharing their personal information over smartphones and social networks based on computer technology and the internet [20].

## Methods

The present qualitative study employed grounded theory. Since the methods that reflect the participants’ views about different phenomena can present more in-depth data, this study aimed to examine the causes and factors affecting violence behaviors among Iranian adolescents using a qualitative methodology. to directly extract the theory from data, which are collected and analyzed based on a specific logic [21]. The data collected from the 40–60 min qualitative interviews conducted in high schools and vocational schools were simultaneously analyzed.

## Participants

The present research used purposive sampling to select 20 male senior high school students with verbally or physically violent behaviors based on their disciplinary profiles or report of informed students.^1^ The participants were selected using purposive sampling and the researchers recruited the participants based on the reports on violent behavior by the teachers, principals and their school portfolios.

## Data collection

The present study was conducted after obtaining the necessary permission and code of ethics from Alborz University of Medical Sciences, Karaj, Iran. The participants were first briefed in person on the study objectives. They were assured of the confidentiality of their information and their voluntary participation. The data were then collected by conducting semi-structured interviews until reaching theoretical data saturation. The interviews were performed by the third author at school. The audio-recorded interviews were then transcribed.

## Data analysis

The findings were obtained in several steps, including conducting interviews, transcription, extracting and merging concepts, forming core categories and creating the main category. To extract causal, contextual and confounding conditions and consequences of violent behaviors from the interview transcripts, the data related to violent behaviors were coded, categorized and analyzed in MAXQDA 2020. Paying more attention to validity than reliability is essential in qualitative research. Lincoln and Guba introduced trustworthiness, credibility, dependability, transferability and confirmability as the criteria for qualitative research [22].

Therefore, the researcher tried not to involve his biases and attitudes into the study and tried to establish rapport with the participants so as to let them express their stories to understand their ideas. In addition to the long-term presence of the researchers in the field, the participants were provided with notes and reports, which were corrected in cases of differences in perceptions and interpretation of data. The present findings were also shared with experts and improved by discussing the codes and categories in several meetings.

Triangulation was used in measurements to reduce the skewed responses of the subjects with violent behaviors, increase the validity of the theoretical statements and consider multiple perspectives [23]. In addition to interviewing the participants with violent behaviors, their disciplinary profiles were therefore reviewed. To collect further information as complementary data from different sources and ensure its validity, informed students and parents of the participants were interviewed. Efforts were made to prevent the researchers’ assumptions from interfering the interviews, and sympathetic introspection was performed to clarify the psychological concept of violence behaviors from the participants’ perspective. The constructs and findings were therefore obtained based on the interviewees’ statements.

## Findings

Twenty students aged 15–19 years with a mean age of 17 and including 12 with violent behaviors and 8 informed cases were included in this study. Eleven participants studied in technical/vocational fields and 45% in theoretical fields. Seven participants studied in the 10^th^ grade, seven in the 11^th^ and six in the 12^th^.

After transcribing and screening all the interviews at their end, 63 open codes (concepts), then 16 core codes (categories) and ultimately a selective category were extracted. The findings of this sections were obtained based on the coding paradigm of Strauss and Corbin [21]. Since this model includes the main theme and different causal, intervening, contextual, strategic elements, it can present an appropriate theoretical framework for understanding the topic under study.

## Causal conditions

Causal conditions refer to events that denote the occurrence and development of a phenomenon [21]. This study identified “economic challenges facing the family”, “academic apathy”, “seeking pleasure”, “self-other differentiation” and “family disorganization” as causal conditions.

### Economic challenges facing the family

Analyzing the findings showed that living conditions and economic problems constituted the main cause of school violence by students. In fact, most of the students, especially the middle and lower class of the community, had to support themselves by doing paid work such as working in restaurants and other service sectors; for instance, Tohid,^2^ who had violently treated his classmates and school officials, said:

“I have to work until 2 midnight to cover my expenses, I’m always sleepy in the classroom, so I had had quarrels with teachers many times; of course, I told them my conditions and they understood.”

Livelihood challenges facing families were therefore found to cause concerns in the participants and reduce their tolerance threshold. Instead of pursuing education as their main mission, the high school students had no choice but to enter the labor market to provide for themselves and their families. They therefore faced numerous sufferings, including violence. The results indicate that some of the students are concerned with financial issues at this age which lowers their social resilience and leads to violent behavior.

### Academic apathy

According to the present findings, the students failed to clearly comprehend education and its effect on their destiny and were forced to select their field of study in some cases. Witnessing numerous cases of inappropriate behaviors by his classmates, Omid said:

“Most of my classmates are not interested in their field and don’t even want to study, because their parents or the education system has selected the field for them”.

According to the findings, the lack of objective and motivation in education constituted the main cause of harassing others at school. Students who are interested in education and their lessons and school lay plans to achieve their educational goals and avoid engaging in violent behaviors.

### Seeking pleasure

Achieving pleasure and happiness affected the tendency towards violent behaviors. The findings suggested the participants committed violence as a way of having fun, taking risks and attracting attention. In other words, they mostly sought pleasure by harming others.

Kian, with a good academic performance, said:

“Perhaps, they think it’s very interesting, it makes them feel good doing it, or they might think their behavior is not bad”.

With divorced parents, Darab said:

“Sometimes my friends ask me the reason for doing these things. My answer is that I calm down and they causes my joy and pleasure”.

The students appeared to seek joy and pleasure by harming and behaving violently toward others.

### Self-other differentiation

The students were found to seek to achieve a superior identity among their classmates and school students by bullying and coercion.

With the experience of fight with students, Afshin said:

“Some people resort to violent behaviors to prove their power. Most of them are arrogant and think they will be a big shot doing that. As the saying goes, they are swollen-headed and immature”.

As a diligent student with strong social communication, Jalal said:

“Adolescents are keen for excitement and tend to introduce themselves as a well-known individual to the community. Lots of people tend to highlight themselves in a group even at the expense of doing bad things. No one can stop them, because they like it, it’s their choice and they like to be well-known”.

Some of the participants appeared to seek a superior and distinct image and identity through violence given their inability to achieve their desired fame in the correct and principled ways.

### Family disorganization

As an entity, the family was found to be incapable of performing its function as a result of facing different livelihood and sociocultural developments and to have neglected many of its duties, especially in terms of proper upbringing of children.

With a history of violent behaviors at school, Masoud explained parental supervision:

“My family are unaware of my violent behaviors at school. The same is true for my friends. They are not blamed in their family. I did not see my friends come and speak about the way they are treated at home”.

Being upset about the behavior of his classmates, Amir Reza said:

“Parents do not take responsibility for their children; they have left them to do whatever they want. Restrictions should be reasonable, neither too strict nor rare”.

The findings showed the diminished role of the family as the main training entity responsible for the socialization of children. The individualism created by development of cyberspace has also attenuated parent–child relationships and family intimacy.

## Contextual Conditions

Contextual Conditions refer to specific situations in which actions/interactions are performed [21]. This study found “school inefficiency”, “environmental conditions” and “cultural diversity” to constitute the contextual Conditions.

### School inefficiency

The schools were found to lack the necessary efficiency, attraction and excitement for the students. Schools are expected to have the necessary dynamism and vitality in different educational dimensions, including training, sports and extracurricular activities. Boring schools and classes increase abnormal and violent behaviors among unmotivated students.

Jafar, an aggressive student with recently divorced parents, explained his negative attitudes toward teachers:

“Teachers do not communicate with students; their classrooms with a strict atmosphere cause students to lose interest in lessons, because everything is based on grades and exams. Of course, some teachers directly talk to and guide students”.

Hussein explained the lack of supervision in school:

“The lack of supervision has increased violence and annoying behaviors in schools. Some students may abuse this neglect and use their force to harass others. School officials take no responsibility for the students’ behavior”.

According to the present findings, the education system should be improved in terms of curricula, manpower and facilities to create positive attitudes towards schools among students.

### Environmental conditions

According to the research findings, living in areas with socially-damaging effects paves the way for committing violent behaviors. Environmental conditions therefore promote violence among residents and cause the spread of this behavioral trait to other environments, including schools.

Shayan, a student with annoying behaviors, said:

“It is also related to the culture of the neighborhood; for example, in our neighborhood, most of my peers are nervous and violent and get angry quickly; nevertheless, I believe residents in poor neighborhoods are loyal compared to wealthy people”.

The behavioral patterns of an area and social environment affect its residents, especially adolescents who tend to learn social behaviors. Migration and population instability in areas such as Karaj with a high frequency of social damage and a violent culture increase tendency to violence.

### Cultural diversity

Cultural differences among the students laid the foundations for their conflicts and anti-social behaviors. Violence can be triggered in a group of adolescents with different socio-cultural perspectives and backgrounds. With a history of several fights at the school, Parsa said:

“There are lots of fights, especially in our school, which is located in a district of Karaj with a low social class. I was unintentionally engaged in several fights at school. A student was looking for trouble. They were somehow insulting my personality”.

As a witness to numerous school conflicts, Sobhan said:

“Difference in opinion and understanding is a common factor among the many. One gets angry and begins insulting by hearing things that contradict their beliefs”.

Different beliefs and lifestyles can lay the groundwork for different understandings of social phenomena, which causes social conflicts in environments with different Iranian subcultures. These differences are reflected as violence behaviors in adolescents at school.

## Confounding conditions

Confounding Conditions refer to structural conditions that belong to a phenomenon, affect actions/interactions and facilitate or restrict strategies within a particular context [21]. The present study identified “peer pressure” and “puberty” as the confounding Conditions.

### Peer pressure

Friendly communication and interactions with individuals with certain behaviors play a key role in forming peer behavior. Carefully selecting friends therefore constitutes a fundamental strategy in establishing healthy and constructive relationships. Regarding the effect of friendly relationships with individuals who have abnormal behaviors, Parviz said:

“We become friends, and over time, as the relationship deepens, we repeat the behaviors of our friends, and this is the case”.

With ample knowledge about the behaviors of his classmates, Mahmoud argued:

“The school atmosphere causes students to support the behavior of their friends; otherwise, they are insulted and harassed”.

According to the participants, friends act as a role model depending on the quality and strength of their relationships.

### Puberty

Puberty, naivety and emotionality cause violent reactions in social interactions.

Mohsen said:

“Easy access to the Internet and information and the lack of parental supervision have caused precocious puberty as a reason for this problem (harsh and emotional behaviors)”.

With violent reactions to others, Reza said:

“We are very weak in the face of problems; for example, the teacher takes a difficult exam, and when we get tired of studying, we react with objections, sadness and frustration. Perhaps, it is our age and naivety that cause these problems”.

According to the conducted interviews, this type of interaction with others and quick reactions are rooted in decreased social resilience and increased irritability, which is noticeable in the face of unfavorable situations. As a psychological trait in adolescents, these reactions manifest themselves as violent behaviors in public settings such as schools.

## Strategy

Strategies are adopted to control, manage and deal with phenomena under certain observed conditions [21]. Interactions, strategies and processes refer to the interactions and actions that actors take in response to those conditions. This study identified the following strategies in the violent adolescents:

### Ignoring school rules

Unmotivated students tend to violate the norms and disobey the rules and regulations of the school, which causes their abnormal behaviors.

### Violence against violence

One of the strategies that students with violent behavior use, particularly when they are not motivated, is to neglect school regulation and commit violence; therefore, they unusual acts when interacting with others.

Adolescents use force and threats to achieve their desires. They may pick a fight to demonstrate their superior position.

Violence in adolescents causes harmful consequences for the individual and negatively affects the community. At all stages and areas of their life, violent individuals resort to violence to resolve tensions and conflicts, which perpetuates the cycle of violence.

## Consequences

As the outcomes of interactions and strategies, consequences are influenced by the conditions associated with interactions. Individuals falling into this cycle have to face “humiliation”, “punishment”, “loss of ideal opportunities in life” and “rejection”. Depending on familial circumstances, individuals affected by the environment and group of friends continue violence and create an insecure social environment.

### Humiliation

As a consequence of violent behaviors, humiliation can be performed by school officials, parents and classmates.

With the experience of these outcomes, Amir Hossein said:

“In response to my behavior that caused harassment to others, they belittled me or asked why I did that while yelling at me”.

Amir Reza said:

“I witnessed the loss of status in students who committed bad behaviors towards their classmates and even the school staff; no one would respect and talk to them. Coming to school, their families faced the same reactions”.

### Loss of ideal opportunities in life

As a consequence of abnormal behaviors in school, loss of ideal opportunities in life disrupted the students’ socialization and deprived them of the necessary skills required to be learned for their future.

With a history of inappropriate behaviors at school, Reza said:

“With these behaviors and actions, our friends and I will ruin our future and fail to get a good job”.

### Punishment

The perpetrators received different forms of punishment as a consequence of their violent behaviors in school. Sobhan, a member of the school’s student council with a history of attended disciplinary meetings, said:

“In the family, they are oppressed by their parents. At school, they are punished by teachers, officials, etc.”.

With a history of violent behaviors at school, Masoud explained the consequences he faced:

“The punishments we receive by our school officials are usually verbal and corporal or involve decreasing the discipline score. In severe cases, temporary or permanent expulsion has happened to I and my friends”.

### Rejection

Rejection is another consequence facing individuals with abnormal behaviors.

Kian said:

“At school or in a friendly group, when the person continues their behavior for a while, no one would want to be friends with them. It makes others end their relationships and no longer talk to them. As I remember, one of my classmates broke the nose of another student in a fight, and in the end, his family paid compensation despite their poor economic status. In fact, my friend’s behavior put his family in a very difficult situation”.

The present research found all the violent students to have confronted the consequences cited. According to the informed adolescents, these students faced humiliation, punishment and social exclusion.

## Core category

This study selected the core category after performing several steps, including interviewing, initial coding, screening and selective coding. The core category was indeed created through selective coding, which integrates and refines categories to create a theory. The researchers immersed themselves in the data to find the links among the categories, refine and integrate them and ultimately extract the theory. The core category, including the main theme and all the categories and concepts obtained, was deduced from all the categories and summarized in a few words [21].

**Fig. 1:**
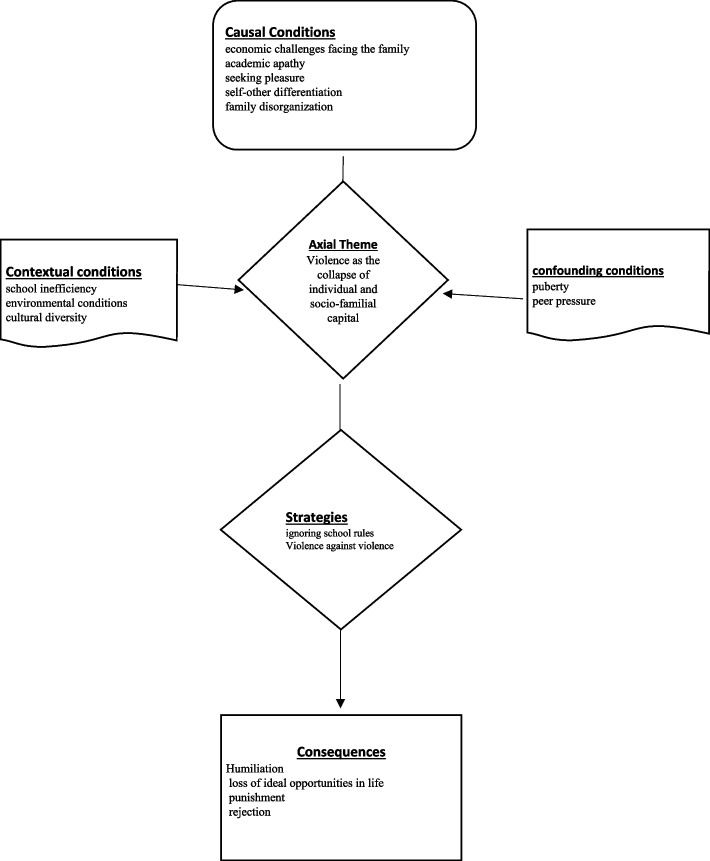
Paradigm model of adolescent violence

“Violence as the collapse of individual and socio-familial capital” was labeled the core category of the present study. This category stands at the most abstract level given that it can analyze the other categories and that violence suggests the collapse of the individual in different situations. Figure 1 shows the paradigm model of violence.

## Discussion and conclusion

As a major issue in schools, adolescent violence behaviors seriously affects the school efficiency. To identify the causes and contexts of this phenomenon, the present study employed semi-structured interviews based on grounded theory to investigate violence in twenty adolescents selected as the witnesses to or perpetrators of violence behaviors in high schools and vocational schools using purposive sampling. To answer “What are the effective factors in adolescent violence?” as the main question, “violence as the collapse of individual and socio-familial capital” was selected as the core category based on grounded theory and the paradigm model. The core category was obtained based on all the analyses and its conceptual relationships with the main categories. The present findings suggested the relationships of adolescent violence behaviors with individual characteristics and familial, socioeconomic, cultural and structural factors. This phenomenon was therefore explained from a comprehensive perspective. Therefore, based on the findings of the present study, the interconnection between the structural factors significantly affects violent behavior among students.

According to the paradigm model, the causal Conditions and driving factors of school violence comprised “economic challenges facing the family”, “academic apathy”, “seeking pleasure”, “self-other differentiation” and “family disorganization”. According to the majority of the interviewees, to gain prestige and a superior position among peers and inner peace and pleasure, even of a false and temporary type, was the main factor that caused violent behaviors given the academic failure and economic and family problems of the perpetrators. The contextual Conditions of violence included “school inefficiency”, “environmental conditions” and “cultural diversity”. The majority of the interviewees cited the lack of motivation and disbelief in the effectiveness of school education in the future of adolescents. Considering school education ineffective in future careers paves the way for academic apathy in students. Dull and boring atmosphere of schools also lays the foundations for violent and annoying behaviors in students.

As confounding conditions, peer pressure and puberty significantly exacerbated violent behaviors. According to the majority of the respondents, behaviors such as bullying and violence affected by adolescence and puberty were performed to fulfill the expectations and desires of friends.

Ignoring school rules and Violence against violence constituted the violent strategies adopted by the adolescents to form the lifestyle they had selected at their age. Violent behaviors are normally committed when school norms and rules are violated and a bullying identity is formed in individuals.

The consequences extracted from the text of the interviews and mentioned by the majority of the participants included humiliation, loss of ideal opportunities in life, punishment and social exclusion. In contrast to the factors that facilitated violence, concerns over the future of life and unpleasant situations such as disciplinary punishments, loss of friends and being deprived of companionship with successful people were identified as the consequences of violence. The present findings are consistent with those obtained by Moreno et al. (2021) [15] on social exclusion, Svanoviana et al. (2019) [15] on disorganized neighborhoods and livelihood problems, Vazina et al. (2015) [19] on the experience of domestic violence, Amama and Ronnen-Shenhaw (2013) [18] on family breakdown and Tavabieh and Al-Ruf (2010) [11] on inefficiency of school staff and use of violent behaviors.

According to the present findings, violence behaviors in adolescents is affected by a multilevel mechanism based on social relationships. As a major obstacle to educational objectives, the violence behaviors emerging at school as a reliable and safe environment can spread to other social domains in the long run. Comprehensive education and care against social damage are therefore essential for decreasing violence behaviors among adolescents.

Because the violent behavior is observed more among the students who are less motivated, get lower grades, and are lower in terms of their academic performance, devising plans to imoprove the quality of education among these students seems necessary. Such plans can positively contribute to decreasing violence behaviors rate in schools. Based on the findings of the study, it seems necessary for different social pathologists and social workers at different levels of personal, family and organizations, and decision making to get involved in the efforts to control violence behaviors in schools. It is also suggested that future research explore the experts judgements and ideas about violence behaviors to understand the solutions for this social issue. This study has some limitations as well. since this was a qualitative research study, there are always concerns about the accuracy of the analysis as the qualitative studies are always susceptible to researcher interpretation biases. Another limitation of the current study is that the statistical sample is limited only to boys. In order to understand adolescent violence, it is necessary to conduct further research among boys and girls to provide a better picture of adolescent violence behaviors in the country.

## Data Availability

The datasets generated during and/or analyzed during the current study are available from the corresponding author on reasonable request.

## Footnotes

1 - Students with comprehensive knowledge about the causes and consequences of the violent behaviors of their classmates.

2 - Pseudonyms are used throughout the study.

## References

[1] Varela JJ, Zimmerman MA, Ryan AM, Stoddard SA, Heinze JE (2021). School attachment and violent attitudes preventing future violent behavior among youth. School attachment and violent attitudes preventing future violent behavior among youth.

[2] http://www.jahannews.com/news/569623/. 2017.

[3] Méndez I, Jorquera AB, Ruiz-Esteban C, Martínez-Ramón JP, Fernández-Sogorb A (2019). Emotional intelligence, bullying, and cyberbullying in adolescents. Int J Environ Res Public Health.

[4] Organization WH (2020). Global status report on preventing violence against children 2020.

[5] Atkinson T. Young Adult’s Feelings of Safety after Observing Physical Violence as Rural Adolescents: A Generic Qualitative Study (Doctoral dissertation). USA: Capella University; 2019.

[6] Varela JJ, Sirlopú D, Melipillán R, Espelage D, Green J, Guzmán J (2019). Exploring the influence school climate on the relationship between school violence and adolescent subjective well-being. Child Indic Res.

[7] Ruiz-Hernández JA, Moral-Zafra E, Llor-Esteban B, Jiménez-Barbero JA. Influence of parental styles and other psychosocial variables on the development of externalizing behaviors in adolescents: a sytematic review. Eur J Psychol Appl Legal Context. 2019.

[8] Álvarez-García D, Núñez JC, García T, Barreiro-Collazo A (2018). Individual, family, and community predictors of cyber-aggression among adolescents. Eur J Psychol Appl Legal Context.

[9] Mayer MJ, Nickerson AB, Jimerson SR (2021). Preventing school violence and promoting school safety: contemporary scholarship advancing science, practice, and policy. Sch Psy Rev..

[10] Tobin AN. A study of the impact of crime shows on contemporary criminal investigations: (Doctoral Dissertation). The George Washington University; 2016.

[11] Thawabieh AM, Al-rofo MA (2010). Vandalism at boys schools in Jordan. Int J Educ Sci.

[12] Ssewanyana D, Van Baar A, Mwangala PN, Newton CR, Abubakar A (2019). Inter-relatedness of underlying factors for injury and violence among adolescents in rural coastal Kenya: A qualitative study. Health Psychol Open.

[13] Ingram JR, Patchin JW, Huebner BM, McCluskey JD, Bynum TS (2007). Parents, friends, and serious delinquency: An examination of direct and indirect effects among at-risk early adolescents. Crim Justice Rev.

[14] Siegel LJ. Criminology: Theories, patterns, and typologies. Boston: Cengage Learning; 2015.

[15] Brazill-Murray CM (2018). Adolescent perceptions of addiction: a mixed-methods exploration of Instagram hashtags and adolescent interviews.

[16] Strøm IF, Thoresen S, Wentzel-Larsen T, Dyb G (2013). Violence, bullying and academic achievement: A study of 15-year-old adolescents and their school environment. Child Abuse Negl.

[17] Alshammari AA. The Role of Socioeconomic Status, Strain, Parental, and Peer Influence on Delinquency among African-American Youth (Doctoral Dissertation). Howard University; 2017.

[18] Hamama L, Ronen-Shenhav A (2013). The role of developmental features, environmental crises, and personal resources (self-control and social support) in adolescents' aggressive behavior. Aggress Violent Beh.

[19] Vézina J, Hébert M, Poulin F, Lavoie F, Vitaro F, Tremblay RE (2015). History of family violence, childhood behavior problems, and adolescent high-risk behaviors as predictors of girls’ repeated patterns of dating victimization in two developmental periods. Violence Against Women.

[20] García-García J, Ortega E, De la Fuente L, Zaldívar F, Gil-Fenoy MJ (2017). Systematic review of the prevalence of school violence in Spain. Procedia Soc Behav Sci.

[21] Strauss A, Kurbin J. Principles of Qualitative Research Methodology: Basic Theory, Procedures and Practices. Translated by Boyouck Mohammadi. Tehran (Iran) Institute for Humanities and Cultural Studies Press, First Edition (In Persian). 2006.

[22] Lincoln YS, Guba EG. Naturalistic inquiry. London: Sage; 1985.

[23] Mays N, Pope C (1995). Qualitative research: rigour and qualitative research. BMJ.

